# Anesthetic Management of Patients With Morvan Syndrome: A Case Report and Literature Review

**DOI:** 10.7759/cureus.58762

**Published:** 2024-04-22

**Authors:** Sarah Oliveira, Adriana Prezado, Beatriz Soares, Miguel Ferreira

**Affiliations:** 1 Anesthesiology, Unidade Local de Saúde Santa Maria, Lisbon, PRT; 2 Anesthesiology, Unidade Local de Saúde Alentejo Central, Évora, PRT; 3 Anesthesiology, Instituto Português de Oncologia de Lisboa Francisco Gentil, Lisbon, PRT

**Keywords:** laryngospasm, local anesthetic, neuromuscular blockade, risk of malignant hyperthermia, dysautonomia, anesthesia management, morvan's syndrome

## Abstract

Morvan syndrome is a rare condition distinguished by hyperactivity within the central, autonomic, and peripheral nervous systems. Due to the limited number of cases, this presents clinical challenges stemming from the scarcity of published literature. We present a successful anesthetic approach for a patient diagnosed with Morvan syndrome scheduled for elective major intra-thoracic surgery to remove metastases from a thymoma. The patient had previously undergone thymectomy, with the syndrome being diagnosed only one year after the surgery. Additionally, we conducted a literature review on the anesthetic management of this condition.

## Introduction

Morvan syndrome is a rare condition primarily diagnosed through clinical evaluation. It is frequently linked with autoantibodies targeting voltage-gated potassium channel complexes (VGKCs). The typical manifestations encompass hyperexcitability of the central nervous system (including neuropsychiatric features such as insomnia, confusion, amnesia and hallucinations), the peripheral nervous system (involving neuromyotonia and neuropathic pain), and the autonomic system (characterized by dysautonomia with symptoms like hyperhidrosis and cardiovascular instability) [1-3]. Due to the rarity of this syndrome, there are only three documented cases in the literature detailing the anesthetic management of Morvan syndrome [4-6]. Herein, we present a case illustrating a successful anesthetic approach and undertake a comprehensive literature review examining the anesthetic implications of this syndrome.

## Case presentation

A 53-year-old man diagnosed with Morvan syndrome was scheduled for elective major intra-thoracic surgery. The patient underwent thymectomy combined with radiotherapy six years earlier due to a thymoma. The diagnosis of Morvan syndrome was established only one year after the thymectomy when the patient began experiencing symptoms such as insomnia, hallucinations, muscle hyperexcitability, hyperhidrosis, and palpitations. Positive tests for leucine-rich glioma-inactivated 1 (LGI1) and contactin-associated protein-like 2 (Caspr2) antibodies were obtained. However, the patient was not tested for anti-acetylcholine receptor (Anti-AChR) antibodies, which are relevant in the context of the use of neuromuscular blockade drugs. Intravenous immunoglobulin therapy as well as corticosteroid treatment were administered, resulting in symptomatic improvement. 

One year before our intervention the patient had a symptomatic resurgence marked by excessive sweating, insomnia, and neuromyotonia, which subsequently improved after receiving five days of immunoglobulin therapy. A positron emission tomography (PET) scan revealed pleural metastases, prompting six cycles of chemotherapy with carboplatin plus paclitaxel. Imaging studies showed persistent metastases after the chemotherapy, leading to the proposal for surgical removal of the metastases.

At the time of our surgery, the patient was under neurology consultation and receiving 400 mg/day of carbamazepine due to neuromyotonia. Additionally, he had hypertension as well as tachycardia and was being followed up in the cardiology clinic, receiving treatment with bisoprolol 5 mg once a day. Electrocardiographic evaluation and Holter monitoring revealed occasional supraventricular and ventricular extrasystoles. Furthermore, he had insulin-treated type 2 diabetes and was under statin therapy for dyslipidemia. An anesthesiology consultation was performed before surgery and recommendations were given to continue all medications perioperatively. With a prophylactic purpose and upon recommendation of the attending neurologist, a course of immunoglobulin therapy was administered three days prior to the surgery. 

Upon hospital admission, which occurred on the day before the surgery, the patient was asymptomatic with no recent episodes of hyperexcitability phenomena, changes in sleep patterns, or excessive sweating. The preoperative analytical assessment revealed no abnormalities, including thyroid function.

The patient underwent a left pleurectomy along with decortication performed through a posterolateral thoracotomy. Before anesthesia induction, an epidural catheter was inserted at the T5-T6 level and tested using 3 mL of lidocaine 1.5% with epinephrine 1:200,000. Intravascular or intrathecal placement of the catheter was ruled out. Additionally, an arterial line was inserted for hemodynamic monitoring as well as serial blood gas analysis. The recommendations outlined in the American Society of Anesthesiologists (ASA) Standards for Basic Anesthetic Monitoring were followed. Bispectral Index (BIS) and quantitative monitoring of neuromuscular blockade using train-of-four (TOF) plus post-tetanic count (PTC) were also employed. A left-sided double-lumen endobronchial tube was used in the airway approach. The correct positioning of the tube was confirmed using flexible fiberoptic bronchoscopy.

The induction and maintenance of anesthesia were performed using total intravenous anesthesia with a target-controlled infusion of propofol using the Schnider model for effect-site concentration and titrated to a BIS level between 40 and 60. We did not observe any changes in the usual doses of propofol or alterations in the density spectral array. To attenuate the physiological response to laryngoscopy, we administered 1.25 mcg/kg of fentanyl and 1 mg/kg of lidocaine for analgesia. Analgesic maintenance was achieved through fentanyl boluses, approximately 1 mcg/kg/h, complemented by a 4 mL bolus of ropivacaine 0.2%, followed by an epidural infusion of ropivacaine 0.2% at 4 mL/h. Additionally, 30 mg of ketorolac was administered prior to surgical incision. Rocuronium was used as a muscle relaxant. For intubation, doses of 0.6 mg/kg were administered and after two minutes, a TOF count of 2/4 twitches was observed. During the initial 45 minutes of surgery, multiple boluses totaling 1.9 mg/kg of rocuronium were administered to achieve a TOF count of zero twitches, but a PTC of 10/10. A rocuronium infusion was initiated, requiring doses of 15 mcg/kg/min to maintain a TOF count of 0 twitches. However, the minimum observed PTC was 8/10. Further doses were not increased as they did not limit the surgical procedure.

**Figure 1:**
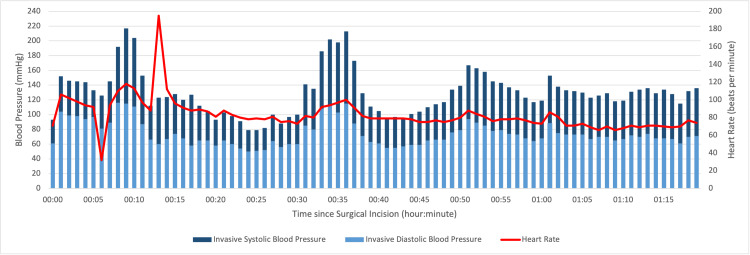
Intraoperative Hemodynamic Parameters

Cardiovascular dysautonomia was observed during the surgery, unrelated to surgical stimuli or depth of anesthesia, and resolved spontaneously without the need for pharmacological intervention (Figure 1). Regarding heart rate, we noted a period of sinus bradycardia and several episodes of sinus tachycardia (ranging from a minimum of 32 beats per minute to a maximum of 195 beats per minute). Furthermore, multiple periods of systolic and diastolic arterial hypertension were also observed.

We observed hyperhidrosis, particularly in the forehead region, despite maintaining the central body temperature measured at the nasopharynx at 36ºC throughout the surgery. We administered 4 mg of dexamethasone and 4 mg of ondansetron for the prophylaxis of nausea and vomiting. The rocuronium infusion was discontinued 20 minutes before the end of the surgery. Based on TOF monitoring showing 2/4 twitches, the reversal of neuromuscular blockade was accomplished with sugammadex 2 mg/kg. An awake extubation was performed without complications.

The patient was transferred to the post-anesthesia care unit (PACU), where he remained for 24 hours after the surgery. Postoperative analgesia was administered using patient-controlled epidural analgesia, which included a basal infusion of 0.2% ropivacaine at a rate of 4-6 mL per hour, along with an additional rescue bolus of 4 mL with a lockout period of 20 minutes. This regimen was complemented with intravenous acetaminophen 1 g every eight hours, intravenous metamizole 2 g every 12 hours, and oral pregabalin 75 mg twice daily. As a rescue analgesic, the patient was prescribed 100 mg of intravenous tramadol every eight hours, as needed. During the time spent in the PACU, two episodes of sinus tachycardia (maximum of 180 beats per minute) and systolic and diastolic hypertension were observed, all spontaneously resolved within less than five minutes and unrelated to pain complaints or stimulation. A similar episode was observed during the physiotherapy session after the patient experienced palpitations, with sinus tachycardia resolving spontaneously as well. No further complications were observed.

## Discussion

Morvan syndrome is a rare condition, with only a few cases reported in the literature and only three of those cases concerning an anesthetic approach [1,3-6]. The etiology remains poorly understood. There have been reports of its emergence following heavy metal poisoning, as paraneoplastic syndromes associated with thymomas in some cases with myasthenia gravis (MG) and other neoplasms (small-cell lung cancer, teratomas, prostate adenoma and carcinoma in situ of the colon). The association with elevated cerebrospinal fluid immunoglobulin G and oligoclonal bands, as well as thyrotoxicosis and autoimmune hypothyroidism, has also been described [1,3].

Supporting the autoimmune etiology of the syndrome, the majority of patients exhibit autoantibodies against VGKCs [1,3]. The most frequently diagnosed antibody targets Caspr2. Some patients exhibit antibodies against LGI1 and a minority of patients present antibodies against contactin-2 [1,3]. Experimental evidence suggests that these antibodies can induce neuronal hyperexcitability by inhibiting voltage-gated potassium outward currents required for the repolarization of the motor nerve [3]. Depending on the type of protein targeted by the antibodies, patients exhibit some phenotypic differences. Characteristics such as the presence of MG, weight loss, and thymomas are frequently associated with patients with anti-Caspr2 antibodies. On the other hand, the syndrome of inappropriate antidiuretic hormone secretion, delirium, mood changes, and myoclonus typically arise in patients with anti-LGI1 antibodies. Contactin-2 is expressed in cardiac conduction tissue. Patients with anti-contactin-2 antibodies have exhibited signs of cardiovascular instability, including tachycardia and alterations in blood pressure [1,3]. Anti-AChR antibodies are also described in patients with thymoma who clinically present with Morvan syndrome but without concurrent MG. An increase in muscular activity, contrary to that observed in MG, was noted [3,7,8]. Anti-N-type calcium channels, antistriated muscle, and antititin antibodies have also been reported in a patient with Morvan syndrome with thymoma [7]. The most frequent symptoms of the syndrome involve the central nervous system including neuropsychiatric features such as insomnia, confusion, amnesia, and hallucinations. Peripheral symptoms commonly include neuromyotonia as well as neuropathic pain. Additionally, autonomic symptoms such as hyperhidrosis, and autonomic instability characterized by arrhythmias and hypertension, are often observed [1,3].

Anesthesia in these patients can be challenging. Neuromyotonia stands out as a main symptom of Morvan syndrome, with a documented case of spontaneous laryngospasm seemingly stemming from the neuromyotonic activity within the laryngeal muscles [9]. As a precaution, we opted to perform an awake extubation of the patient, which proceeded without adverse events.

Cardiovascular instability was one of the challenges we observed in our case. Despite the patient being asymptomatic prior to surgery and continuing their usual beta-blocker medication, as well as receiving prophylaxis with immunoglobulin, they experienced episodes of tachycardia, bradycardia, and hypertension for unexplained reasons, without any triggering factors. These episodes spontaneously resolved without the need for pharmacological therapy. This indicates that regardless of the patient's previous state, we may observe intraoperative dysautonomia, reinforcing the need for invasive blood pressure monitoring in these patients. 

One of the main points of anesthetic management that we observed was the need for increased doses of rocuronium to achieve a TOF of zero twitch. Royston et al. had already mentioned the same difficulty in managing neuromuscular blockade with rocuronium [4], while Tufail et al., using atracurium, did not reference any increased need for neuromuscular relaxant dose [6]. What was observed in common in both patients requiring increased doses of neuromuscular relaxants is the presence of thymoma or thymoma metastases in patients without MG. The increased muscle activity in patients with thymoma but without MG had already been reported by Halbach et al. in patients with anti-AChR antibodies [8]. In our case, these antibodies were not investigated and Royston et al. also did not mention the study of these antibodies [4]. The presence of antibodies anti-AChR in patients with thymoma may explain the increased resistance to neuromuscular blockade, which underscores the idea of investigating these antibodies in these specific patients. Monitoring neuromuscular blockade in these individuals due to its unpredictable behavior is crucial. 

There is some concern about the risk of malignant hyperthermia, although thus far defects in the ryanodine receptor have not been implicated in the syndrome. In our case, total intravenous anesthesia was used, but in the cases of Royston et al. [4], Singh et al. [5], and Tufail et al. [6], maintenance was carried out with sevoflurane without any complications reported. The inherent risk associated with the use of succinylcholine is uncertain since its use has never been described.

The safety of employing local anesthetics remains uncertain. We administered intravenous lidocaine along with epidural ropivacaine, without any complications. Tufail et al. had previously described the use of intravenous lidocaine for analgesia during induction [6]. In that scenario, the patient experienced a cardiorespiratory arrest believed to be of vagal origin and not necessarily related to local anesthetics. However, it cannot be ruled out that there may be an increased sensitivity to local anesthetics.

## Conclusions

Anesthetizing a patient with Morvan syndrome poses a significant challenge for anesthesiologists. Airway management requires consideration of the risk of laryngospasm due to neuromyotonia. Patients who were previously asymptomatic and underwent prophylactic therapy may exhibit hemodynamic instability attributable to a variety of factors, namely dysautonomia, not only during surgery, but also in the postoperative period. Another concern is the potential resistance to non-depolarizing muscle relaxants in some cases, for reasons that remain unclear. Therefore, it is advisable to quantitatively monitor neuromuscular blockade closely. The use of depolarizing muscle relaxants has not been reported and their safety profile is unknown. Additionally, the risk of malignant hyperthermia is uncertain, but the use of halogenated agents like sevoflurane appears to be safe thus far. The intravenous administration of local anesthetics and their use in regional anesthesia were employed without apparent complications; however, the risk of increased sensitivity to them cannot be ruled out.
